# Bayesian Regression Quantifies Uncertainty of Binding Parameters from Isothermal Titration Calorimetry More Accurately Than Error Propagation

**DOI:** 10.3390/ijms242015074

**Published:** 2023-10-11

**Authors:** Van N. T. La, David D. L. Minh

**Affiliations:** 1 Department of Biology, Illinois Institute of Technology, Chicago, IL 60616, USA; vla@hawk.iit.edu; 2 Department of Chemistry, Illinois Institute of Technology, Chicago, IL 60616, USA

**Keywords:** Isothermal Titration Calorimetry (ITC), Bayesian Credible Interval (BCI), Confidence Interval (CI), Asymptotic Standard Error (ASE), Maximum Likelihood Estimation (MLE), Error Propagation (EP)

## Abstract

We compare several different methods to quantify the uncertainty of binding parameters estimated from isothermal titration calorimetry data: the asymptotic standard error from maximum likelihood estimation, error propagation based on a first-order Taylor series expansion, and the Bayesian credible interval. When the methods are applied to simulated experiments and to measurements of Mg(II) binding to EDTA, the asymptotic standard error underestimates the uncertainty in the free energy and enthalpy of binding. Error propagation overestimates the uncertainty for both quantities, except in the simulations, where it underestimates the uncertainty of enthalpy for confidence intervals less than 70%. In both datasets, Bayesian credible intervals are much closer to observed confidence intervals.

## 1. Introduction

Isothermal titration calorimetry (ITC) is widely used to characterize binding processes involving biomolecules, including proteins [1], small organic molecules [2], DNA/RNA [3,4], and lipids [5]. ITC data are routinely analyzed to estimate thermodynamic parameters—the Gibbs free energy ΔG and the enthalpy ΔH—for simple binding processes. Based on the relation ΔG=ΔH−TΔS that includes the temperature T, the entropy ΔS may also be obtained. These parameters have often been estimated using what we will refer to as the standard procedure: a nonlinear least squares regression method implemented in the Origin software package that is distributed with the MicroCal VP-ITC instrument and its successors. The software yields a maximum likelihood estimate (MLE) of the parameters and asymptotic standard error (ASE). Unfortunately, the ASE underestimates the uncertainty by as much as an order of magnitude [6]!

The severe underestimation of uncertainty is mainly a consequence of ignoring the error in the titrant concentration. In the standard procedure, the error in the titrand concentration (in the sample cell) is handled by assigning the stoichiometry *n* as a free parameter. On the other hand, the titrant concentration (in the syringe) is treated as a constant. This assumption is made because ITC data can only be used to estimate *the ratio* of the titrant:titrand concentrations as opposed to individual values [7]. However, it is a poor assumption, because large errors (10–20%) in titrant concentration between laboratories have been observed [6].

In 2015, Boyce et al. suggested that the uncertainty of the standard procedure could be adjusted based on error propagation [8]. Specifically, based on the Taylor expansion, errors in $K_{a}$, ΔH, and the site parameter *n* may be corrected by the relative error of the titrant concentration. However, they did not show that the resulting uncertainty estimate leads to accurate confidence intervals. Confidence intervals are accurate when the X% confidence interval includes the true value X% of the time, where X is a confidence level. In 2018, Nguyen et al. described the analysis of ITC data with Bayesian regression. They found that *Bayesian credible intervals* (BCIs)—regions that contain a specified percentage of the Bayesian posterior—are more accurate *confidence intervals* than those based on the ASE [9]. There was no comparison to confidence intervals based on the ASE augmented with error propagation. The purpose of this short manuscript is to address this oversight.

## 2. Results and Discussion

### 2.1. Bayesian Posteriors Are Converged

As in Nguyen et al. [9], the Markov chain Monte Carlo protocol leads to converged BCIs. In a representative run for one of the 1000 simulated integrated heat curves (Figure S1), fewer than 10% of samples are required before estimated percentiles of the posterior density are stable (Figure S2). Comparable convergence behavior is observed when sampling the Bayesian posterior for the ITC experiments (Figure S3).

### 2.2. Error Propagation Expands Confidence Intervals to Be Larger Than Bayesian Credible Intervals

**Figure 1:**
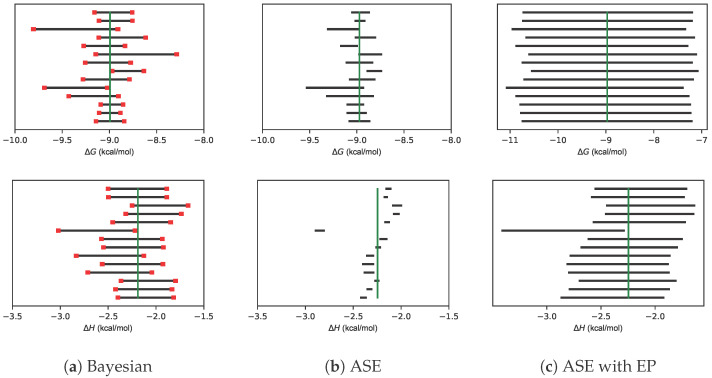
Uncertainty estimates of Mg(II):EDTA dataset. 95% credible intervals estimated from the Bayesian posterior (**a**), confidence intervals calculated by ASE from nonlinear least squares (**b**), and confidence intervals calculated by ASE with EP (**c**) for parameters specifying magnesium binding to EDTA. The median MCMC samples are shown by the vertical green lines. The standard deviations of the lower and upper bounds are denoted as red bars and estimated by bootstrapping.

For each of the 14 experiments, 95% CIs of ΔG and ΔH are shown in Figure 1. Because the true value of parameters is unknown, the median estimate is shown as a proxy. Panels (a) and (b) reproduce Figure 6 from Nguyen et al. [9]. The 95% CIs of ΔG encompass the median value in nearly every experiment. For CIs based on the ASE, 95% CIs of ΔH are too small. Panel (c) shows that error propagation increases CIs to encompass the median, but the CIs appear to be larger than necessary.

### 2.3. Even with Error Propagation, BCIs Provide More Accurate CIs Than the ASE

The accuracy of CIs was more carefully assessed by coverage plots, in which predicted confidence intervals are plotted against the percentage of BCIs and CIs that contain the true values of ΔG and ΔH. For accurate CIs, points should lie along the diagonal. If points are below the diagonal, CIs are underestimated. If points are above the diagonal, CIs are overestimated.

**Figure 2:**
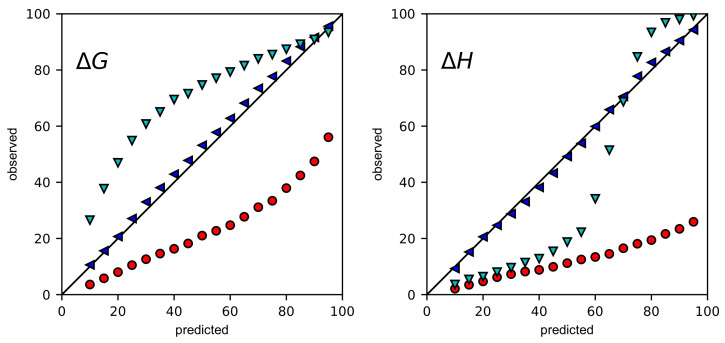
Uncertainty validation of the simulation dataset at high error of 10%. The predicted rate (%) of CIs containing the true values were plotted against the observed rate (%) for Bayesian credible intervals (blue leftward triangles), nonlinear least squares confidence intervals (red circles), and nonlinear least squares confidence intervals with error propagation (cyan downward triangles). Error bars of Bayesian procedure, which were standard deviations based on 100 bootstrapping samples, were too small to be visible.

**Figure 3:**
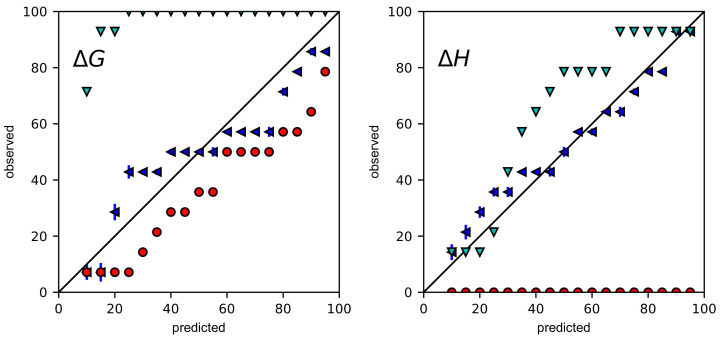
Uncertainty validation of Mg(II)-EDTA dataset. The predicted rate (%) of CIs containing the true values were plotted against the observed rate (%) for Bayesian credible intervals (blue leftward triangles), nonlinear least squares confidence intervals (red circles), and nonlinear least squares confidence intervals with error propagation (cyan downward triangles). Error bars of Bayesian procedure were standard deviations based on 100 bootstrapping samples.

Coverage plots were generated for 1000 simulations with high error (Figure 2) and low error (Figure S4) and for 14 experiments (Figure 3). In all of the coverage plots, Bayesian credible intervals are closest to the diagonal. As expected, the ASE consistently underestimates confidence intervals. For ΔG, the ASE with error propagation overestimates confidence intervals for nearly all confidence levels. For ΔH, the story is more subtle. In the simulations at both high and low error, confidence intervals are underestimated for confidence levels less than 70% but somewhat overestimated for higher confidence levels. In the experiments, confidence intervals are overestimated for confidence intervals greater than 30%.

## 3. Materials and Methods

### 3.1. Integrated Heat Data

Our data are integrated heat curves, $D\in \left\{q_{1},q_{2},\ldots ,q_{N}\right\}$, where $q_{n}$ is the integrated heat of injection *n*. We analyzed simulations as well as ITC experiments that were previously described [9].

Simulations are useful because it is inexpensive to collect large amounts of data and because thermodynamic parameters are known exactly. Simulations of simple 1:1 binding were performed in a similar way as in Nguyen et al. [9]. A total of 1000 integrated heat curves with 24 injections each were modeled based on the free energy of binding ΔG, the enthalpy of binding ΔH, the enthalpy of dilution and stirring per injection $\Delta H_{0}$, the concentration of receptor (titrand) ${\left[R\right]}_{0}$, the concentration of ligand (titrant) ${\left[L\right]}_{s}$, and the standard deviation of the measurement error σ. The thermodynamic parameters and enthalpy of injection were fixed at ΔG = −10 kcal/mol, ΔH = −5 kcal/mol, and $\Delta H_{0}$ = 0.5 μcal; ${\left[R\right]}_{0}$ and ${\left[L\right]}_{s}$ were sampled from lognormal distributions with mean values of 0.1 and 1.0 mM, respectively. Based on the uncertainty of 10% observed by Myszka et al. [6], the variance was set at either small (5% of the mean) or large (10% of the mean). Measurement error was modeled as normally distributed with a zero mean and standard deviation of σ = 1 μcal.

We also analyzed 14 integrated heat curves from previously performed experiments in which MgCl${}_{2}$ was titrated into a sample cell containing EDTA in a MicroCal VP-ITC calorimeter [9].

### 3.2. Regression

Data were analyzed via Bayesian regression and maximum likelihood estimation. In both procedures, integrated heat curves for simple 1:1 binding were modeled as previously described [9]. They are functions of the aforementioned parameters,

(1) $$\begin{array}{c} \theta \equiv (\Delta G,\Delta H,\Delta H_{0},{\left[R\right]}_{0},{\left[L\right]}_{s},\sigma ). \end{array}$$

Observed injection heat $q_{n}$ was treated as normally distributed about the true heat $q_{n}^{*}\left(\theta \right)$,

(2) $$\begin{array}{c} q_{n}∼N\left(q_{n}^{*}\left(\theta \right),\sigma^{2}\right). \end{array}$$

Thus, the likelihood function of an integrated heat curve $D\in \left\{q_{1},q_{2},\ldots ,q_{N}\right\}$ is

(3) $$\begin{array}{c} p\left(D|\theta \right)=\frac{1}{{\left(2\pi \right)}^{N/2}\sigma^{N}}\exp\left[-\frac{1}{2\sigma^{2}}\sum_{n=1}^{N}{\left(q_{n}-q_{n}^{*}\left(\theta \right)\right)}^{2}\right]. \end{array}$$

#### 3.2.1. Bayesian Regression

For the Bayesian regression, the prior of parameters was independent, such that $p\left(\theta \right)=\prod_{i}p\left(\theta_{i}\right)$. As in Nguyen et al. [9], uniform priors were used for ΔG, ΔH, and $\Delta H_{0}$. Lognormal priors were used for the concentrations of the ligand and the receptor,

(4) $$\begin{array}{c} \ln{\left[X\right]}_{0}∼\mathrm{LN}\left({\left[X\right]}_{0},{\left(\delta {\left[X\right]}_{0}\right)}^{2}\right), \end{array}$$

where ${\left[X\right]}_{0}\in \left\{{\left[R\right]}_{0},{\left[L\right]}_{s}\right\}$ is the stated value of each quantity; δ was assumed to be either 5% or 10%. The uninformative Jeffreys prior was used for σ [10]:

(5) $$\begin{array}{c} p\left(\sigma \right)\propto \frac{\sigma_{0}}{\sigma }, \end{array}$$

where $\sigma_{0}=1\;\mathrm{cal}$. For this model, the posterior probability density is

(6) $$\begin{array}{c} p(\theta |D)\propto p(D|\theta )\;p(\theta ), \end{array}$$

Sampling from the Bayesian posterior was performed using a Markov chain Monte Carlo method, as in Nguyen et al. [9], but with a few small adjustments. Instead of using pymc3, the regression was implemented in numpyro [11,12]. After 2000 warm-up moves, 10,000 (as opposed to 5000 [9]) samples from four chains were stored. The X% BCI of each parameter was calculated based on the smallest interval that contains X% of the posterior samples. Additionally, the uncertainty δ was set at either 5% and 10%, as opposed to only 10%.

#### 3.2.2. Maximum Likelihood Estimation

For the MLE, parameter estimates were obtained as

(7) $$\begin{array}{c} \hat{\theta }=\arg\max_{\theta }\log p\left(D|\theta \right). \end{array}$$

The covariance matrix of the asymptotic standard error (ASE) was estimated based on the inverse Fisher information matrix,

(8) $$\begin{array}{c} cov\left(\hat{\theta }\right)\approx -\frac{1}{N}{\left[\frac{\partial logL_{N}}{\partial \theta \partial \theta^{⊤}}\left|{}_{\theta =\hat{\theta }}\right.\right]}^{-1}. \end{array}$$

We used scipy.optimize.minimize function from the python package scipy [13] to implement this MLE model, estimate the parameters, and automatically calculate the covariance matrix for parameter uncertainty. The X% CI of each parameter was defined by an interval in which the lower bound was the 1 − X/2 percentile, and the upper bound was the 1 + X/2 percentile of the normal distribution with a mean as the estimated value and standard deviation as the ASE.

#### 3.2.3. Maximum Likelihood Estimation with Error Propagation

We performed error propagation to augment the ASE of MLE parameters based on the formula provided by Boyce et al. [8],

(9) $$\begin{array}{c} {\left(\frac{s_{\theta }}{\theta }\right)}^{2}={\left(\frac{s_{\theta ,ASE}}{\theta }\right)}^{2}+{\left(\frac{s_{{\left[L\right]}_{s}}}{{\left[L\right]}_{S}}\right)}^{2} \end{array}$$

In this equation, *s* are standard errors and θ∈{ΔG,ΔH} are the parameters affected by the uncertainty of ligand concentration ${\left[L\right]}_{S}$; $s_{\theta ,ASE}$ is the ASE, $s_{{\left[L\right]}_{s}}$ is the standard error in the ligand concentration, and $s_{\theta }$ is the error estimate of the parameter θ that incorporates ligand concentration error. The uncertainty of the ligand concentration $s_{{\left[L\right]}_{s}}$ can be estimated by another experiment or based on previous estimates. Considering uncertainty in both protein and ligand concentrations, we used either 5% or 10% for both $s_{{\left[R\right]}_{0}}/{\left[R\right]}_{0}$ and $s_{{\left[L\right]}_{S}}/{\left[L\right]}_{S}$. CIs of this procedure were estimated similarly to the MLE procedure.

## 4. Conclusions

In both ITC simulations and experiments, BCIs provide more accurate uncertainty estimates for thermodynamic binding parameters than the ASE, without or with error propagation. The ASE underestimates the uncertainties of all datasets. Error propagation overestimates the uncertainties in the experimental dataset, but in simulations it underestimates the uncertainty of enthalpy for confidence intervals less than 70%.

## Data Availability

All code is freely available at https://github.com/vanngocthuyla/bitc/tree/main/bitc_nls_ep, accessed on 30 July 2023.

## Supplementary Materials

The following supporting information can be downloaded at: https://www.mdpi.com/article/10.3390/ijms242015074/s1.
