# Supplementary material for: Bayesian Regression Quantifies Uncertainty of Binding Parameters from Isothermal Titration Calorimetry More Accurately Than Error Propagation
Source: Int J Mol Sci. 2023 Oct 11;24(20):15074. doi: 10.3390/ijms242015074 (PMC10606514; doi:10.3390/ijms242015074)

Supplementary for "Bayesian Regression Quantifies Uncertainty of Binding Parameters from Isothermal Titration Calorimetry More Accurately Than Error Propagation"

Van Ngoc Thuy La[†] and David D. L. Minh[*,‡]

†*Department of Biology, Illinois Institute of Technology, Chicago, IL 60616, USA*

‡*Department of Chemistry, Illinois Institute of Technology, Chicago, IL 60616, USA*

E-mail: dminh@iit.edu

Contents

Figure S1. One thousand simulation curves of 1:1 binding ITC data S2
Figure S2. Convergence analysis of Bayesian sampling for a representative simulated dataset S2
Figure S3. Convergence analysis of the Bayesian posterior for Mg(II)-EDTA datasets S3
Figure S4. Uncertainty validation of the simulation dataset at low error S17

Figure S1. One thousand simulation curves of 1:1 binding ITC data.

Parameters for the curves are in the Materials and Methods section of the main text.

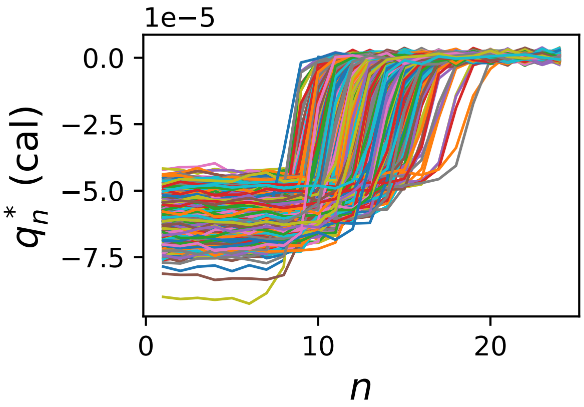

Figure S2. Convergence analysis of Bayesian sampling for a representative simulated dataset.

There were 10,000 samples generated from the Bayesian posterior. All six parameters are shown. The 5th, 25th, 50th, 75th, and 95th percentiles are represented as lines with blue circles, green squares, red diamonds, cyan upward triangles, and magenta downward triangles, respectively. The error bars estimated by 100 bootstrapping samples are too small to be visible.

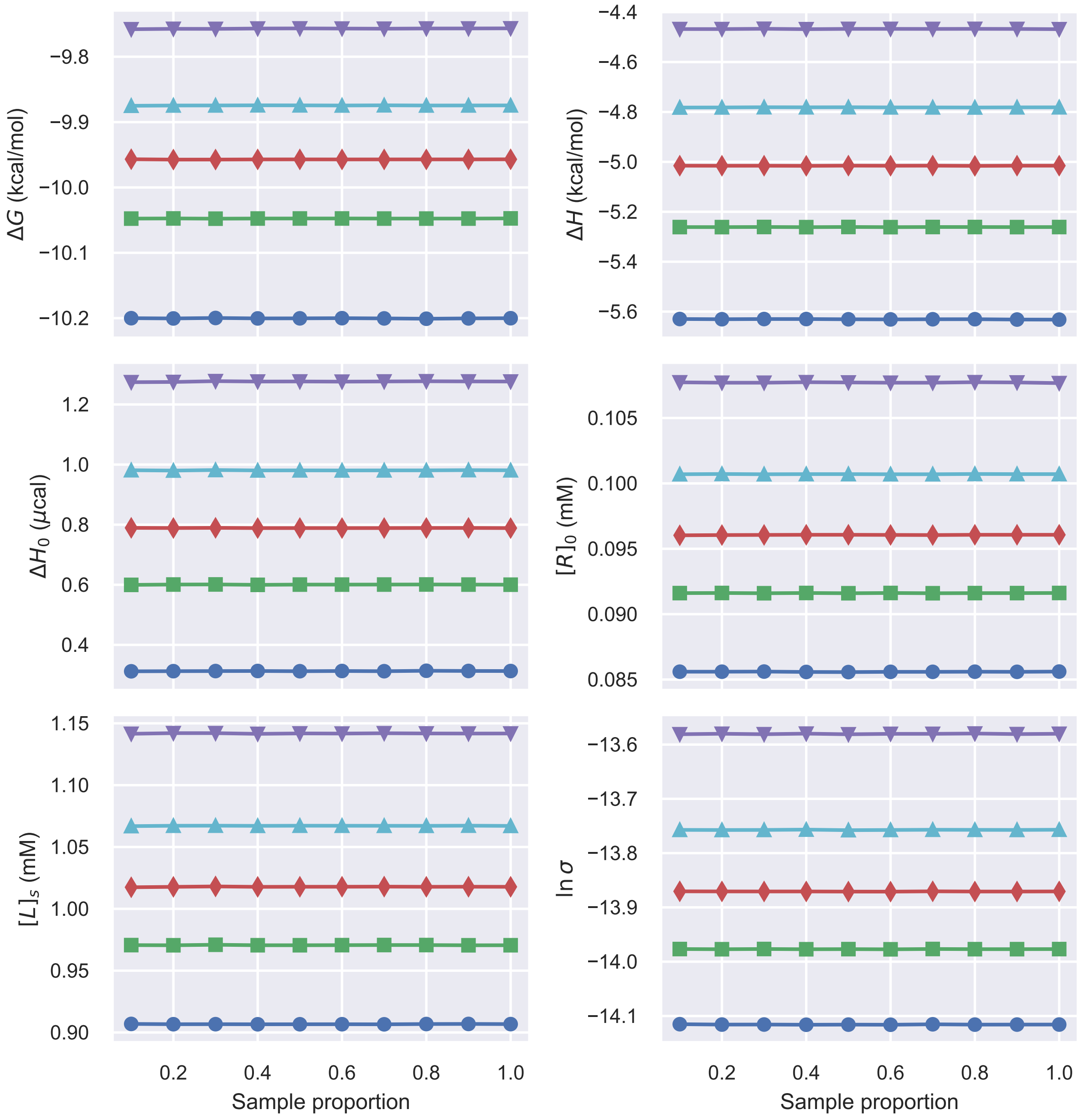

Figure S3. Convergence analysis of the Bayesian posterior for Mg(II)-EDTA datasets.

There were 10,000 samples generated from the Bayesian posterior. All six parameters are shown. The 5th, 25th, 50th, 75th, and 95th percentiles are represented as lines with blue circles, green squares, red diamonds, cyan upward triangles, and magenta downward triangles, respectively. The error bars estimated by 100 bootstrapping samples are too small to be visible.

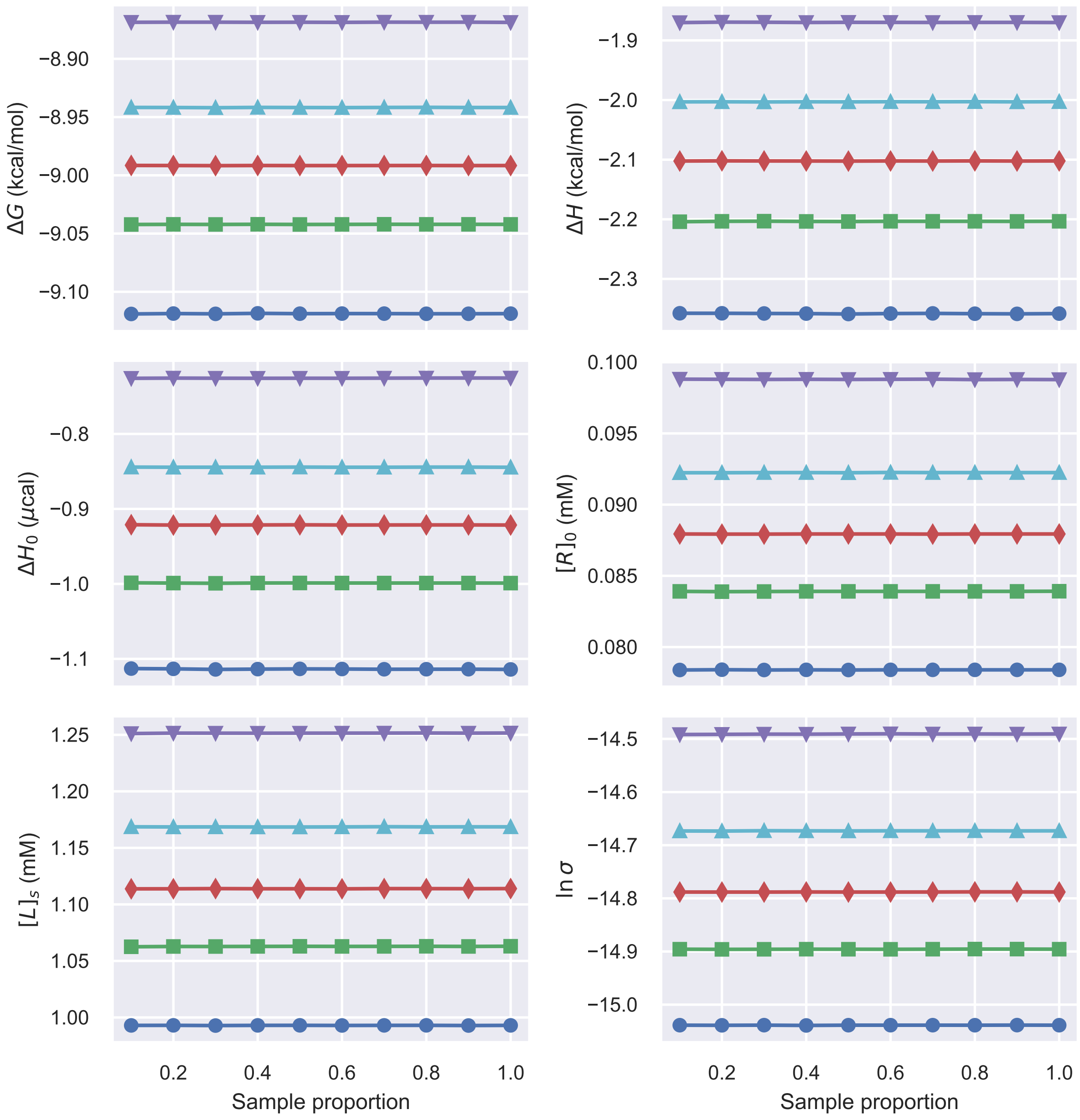

ΔG (kcal/mol)
−8.90
−8.95
−9.00
−9.05
−9.10
ΔH (kcal/mol)
−1.9
−2.0
−2.1
−2.2
−2.3
ΔH0 (μcal)
−0.8
−0.9
−1.0
−1.1
[R]0 (mM)
0.100
0.095
0.090
0.085
0.080
[L]s (mM)
1.25
1.20
1.15
1.10
1.05
1.00
ln σ
−14.5
−14.6
−14.7
−14.8
−14.9
−15.0
0.2
0.4
0.6
0.8
1.0
Sample proportion

Mg1EDTAp1a

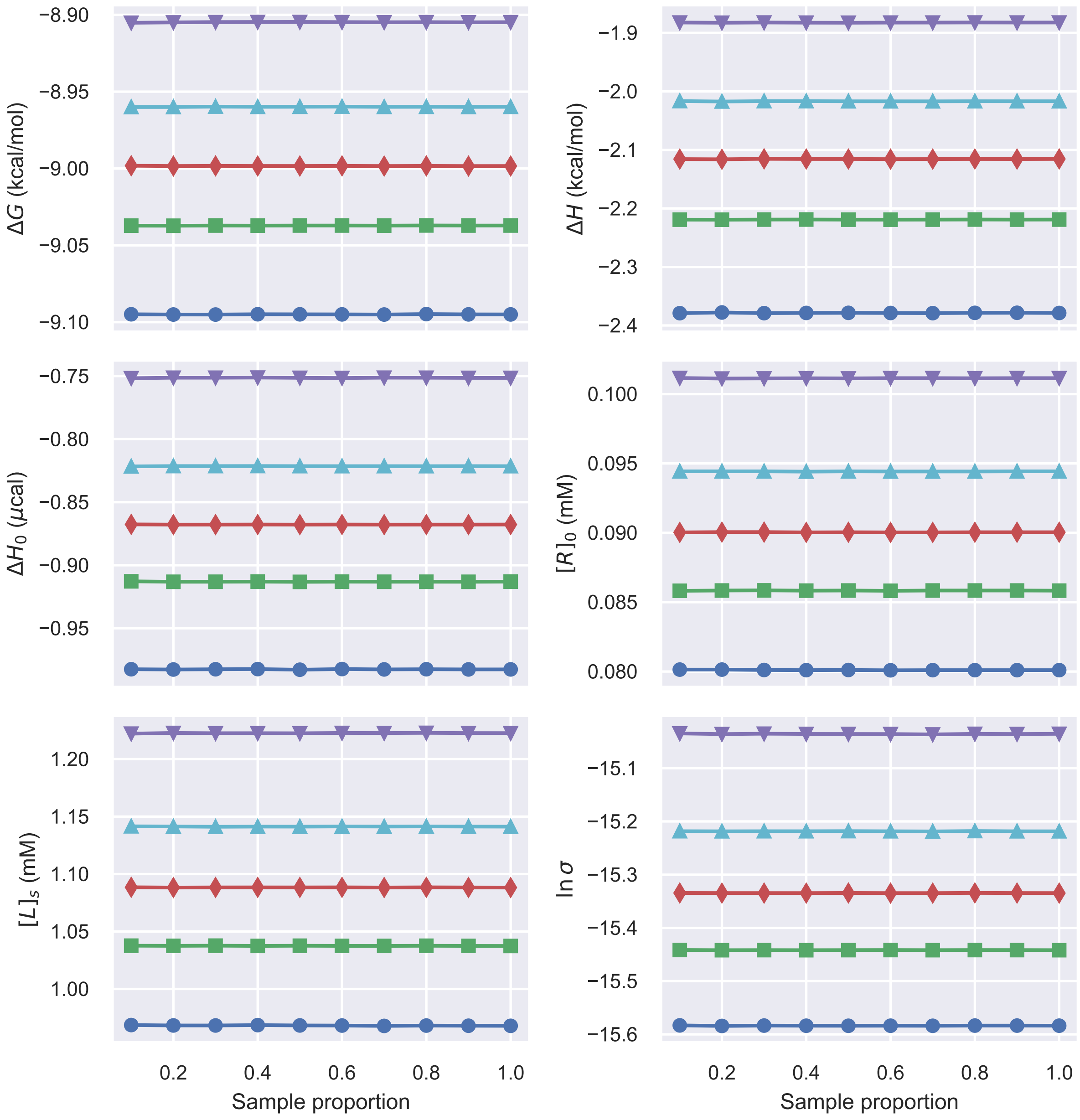

ΔG (kcal/mol)
ΔH (kcal/mol)
ΔH0 (μcal)
[R]0 (mM)
[L]s (mM)
ln σ
Sample proportion
Sample proportion

Mg1EDTAp1b

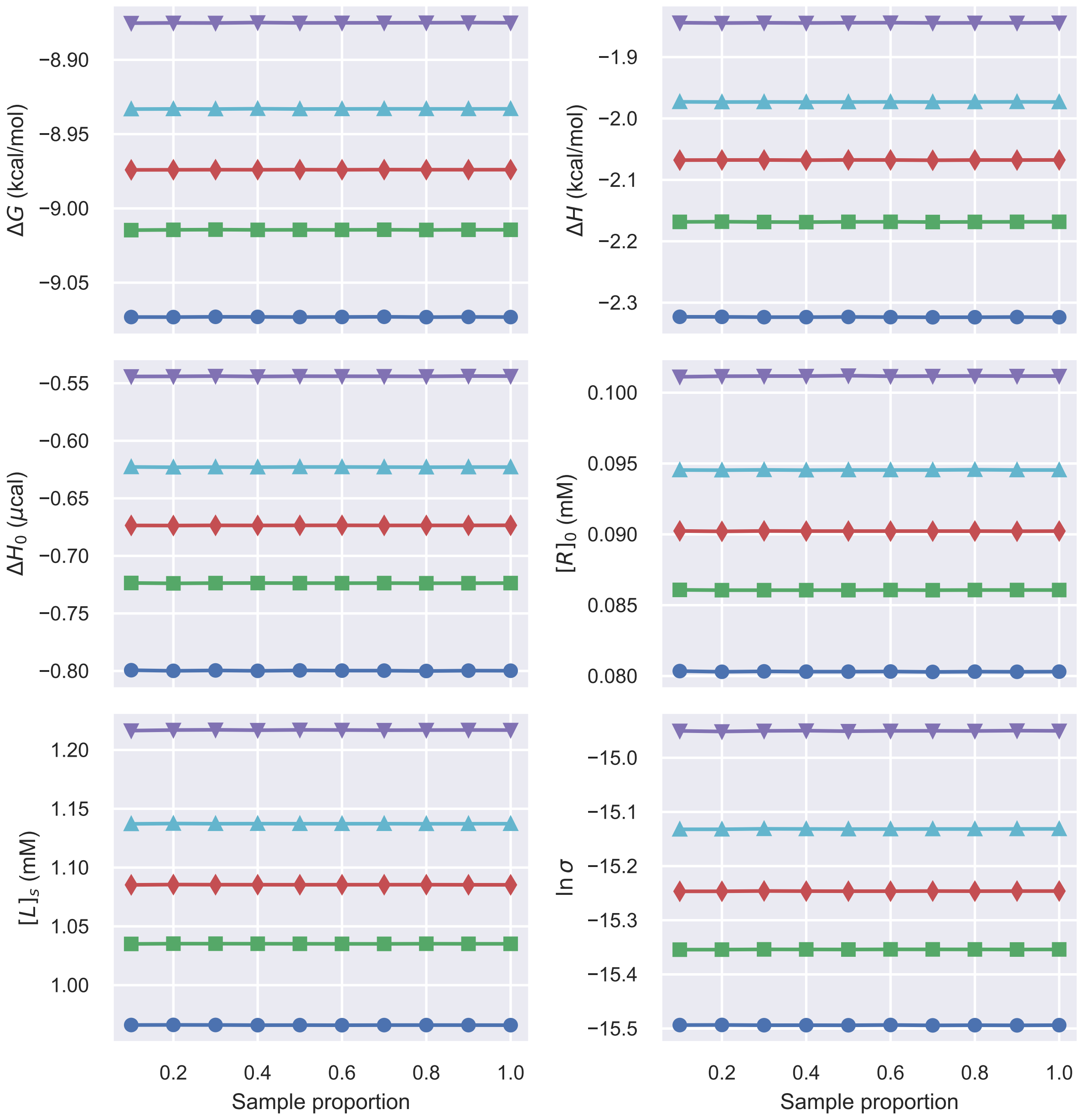
ΔG (kcal/mol)
−8.90
−8.95
−9.00
−9.05
ΔH (kcal/mol)
−1.9
−2.0
−2.1
−2.2
−2.3
ΔH0 (μcal)
−0.55
−0.60
−0.65
−0.70
−0.75
−0.80
[R]0 (mM)
0.100
0.095
0.090
0.085
0.080
[L]s (mM)
1.20
1.15
1.10
1.05
1.00
ln σ
−15.0
−15.1
−15.2
−15.3
−15.4
−15.5
0.2
0.4
0.6
0.8
1.0
Sample proportion
Sample proportion

Mg1EDTAp1c

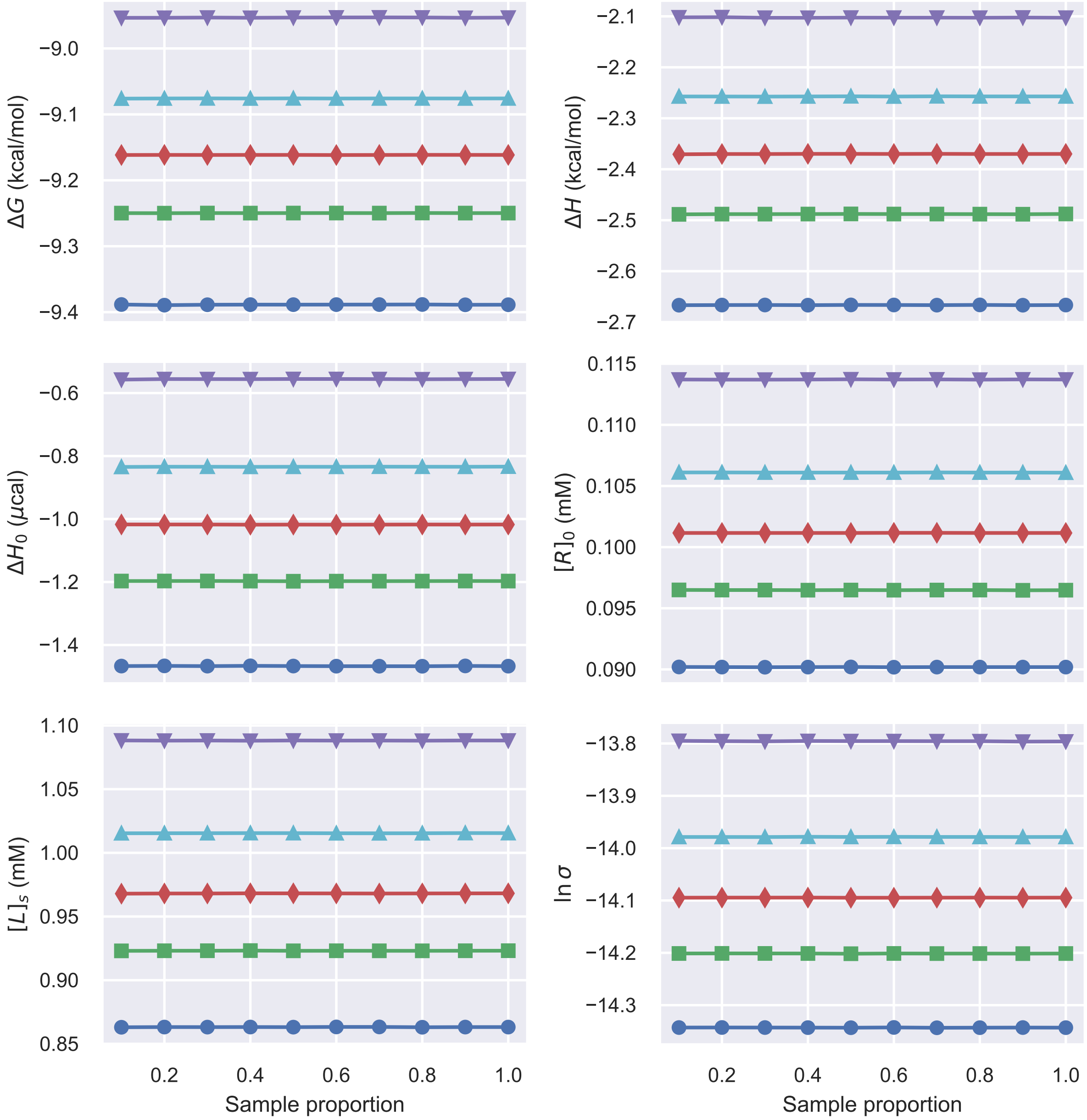

ΔG (kcal/mol)
ΔH (kcal/mol)
ΔH0 (μcal)
[R]0 (mM)
[L]s (mM)
ln σ
Sample proportion
Sample proportion

Mg1EDTAp1d

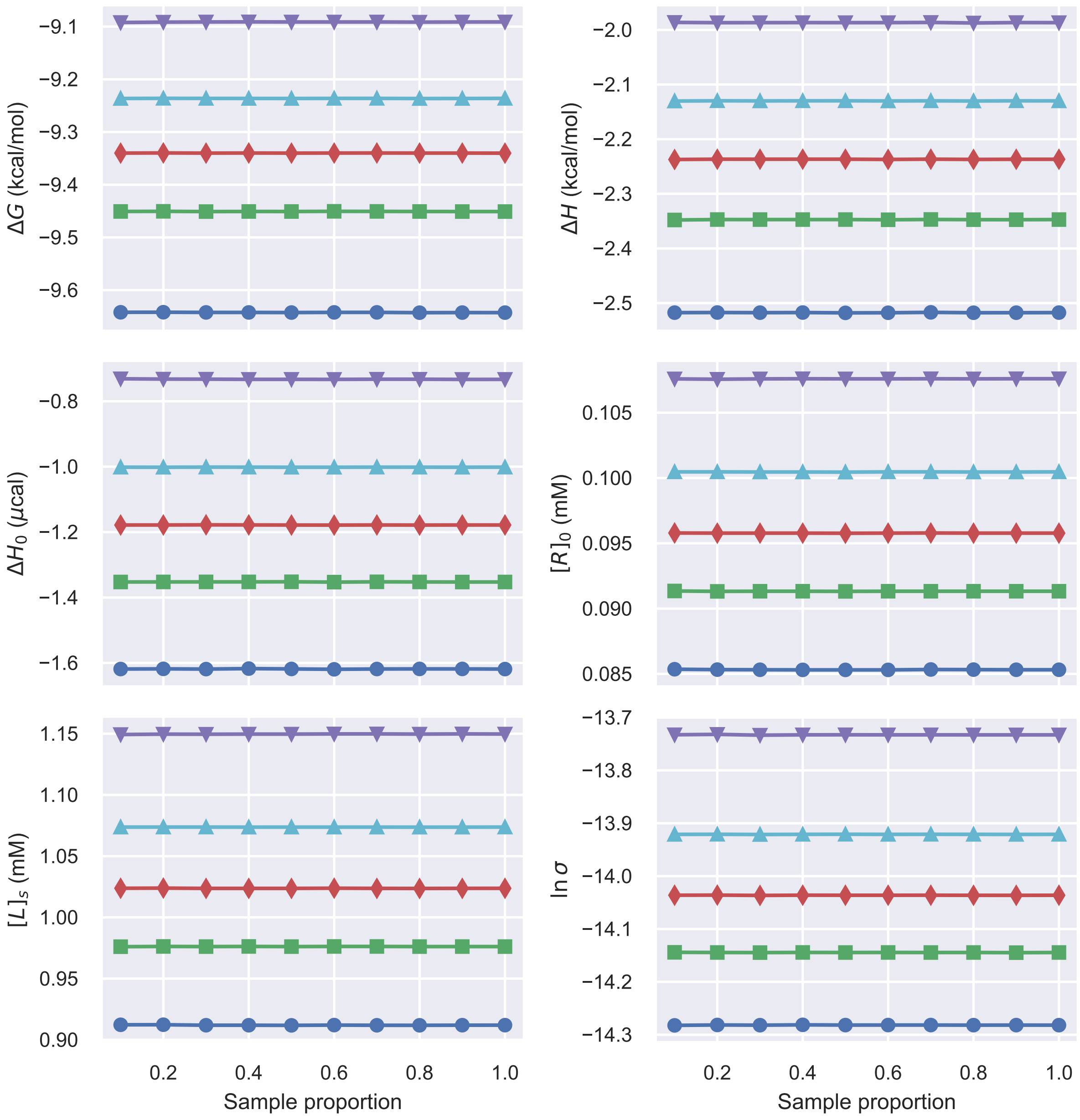

ΔG (kcal/mol)
ΔH (kcal/mol)
ΔH_0 (μcal)
[R]_0 (mM)
[L]_s (mM)
ln σ
Sample proportion
Sample proportion

Mg1EDTAp1e

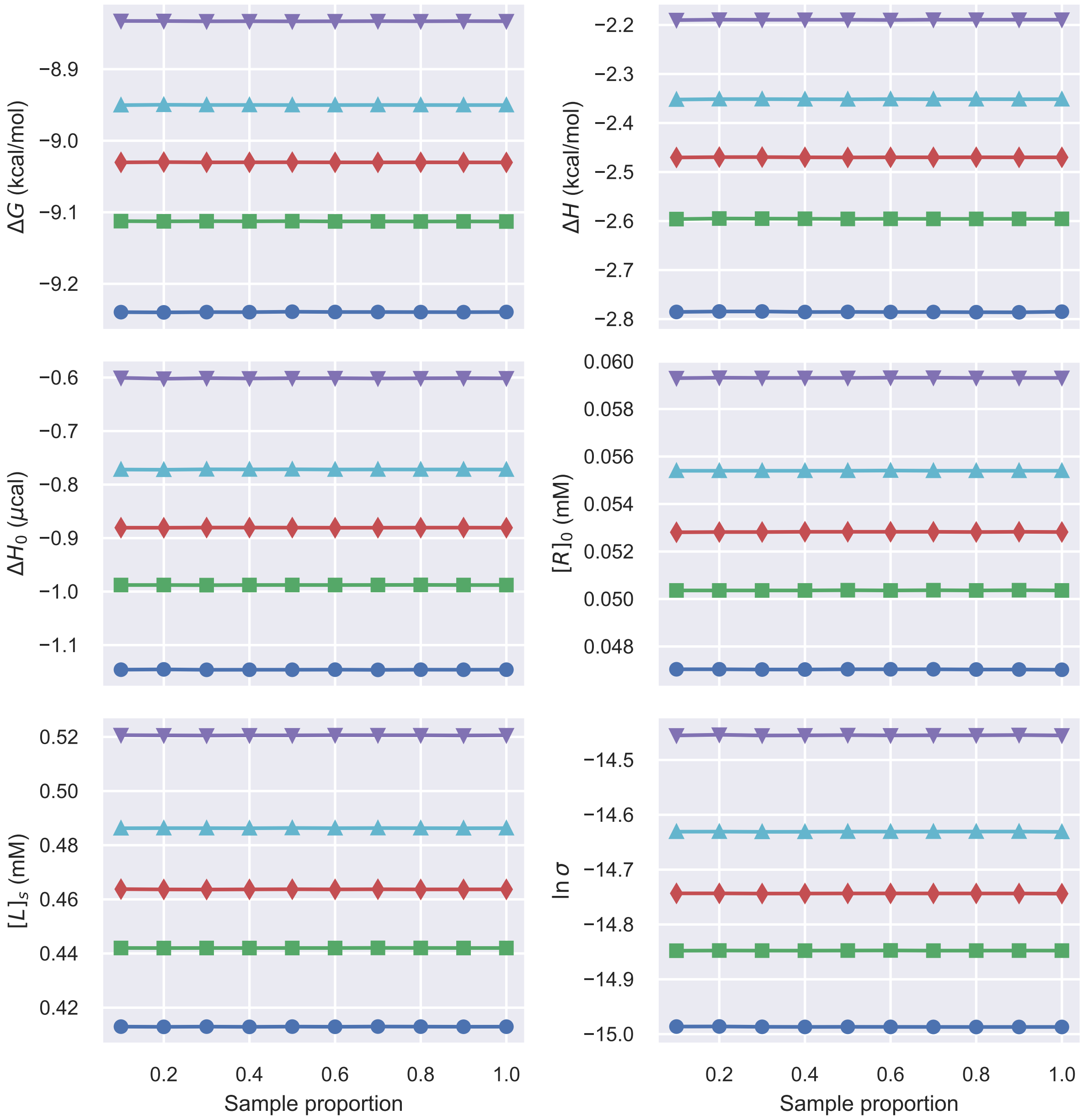

Mgp5EDTAp05a

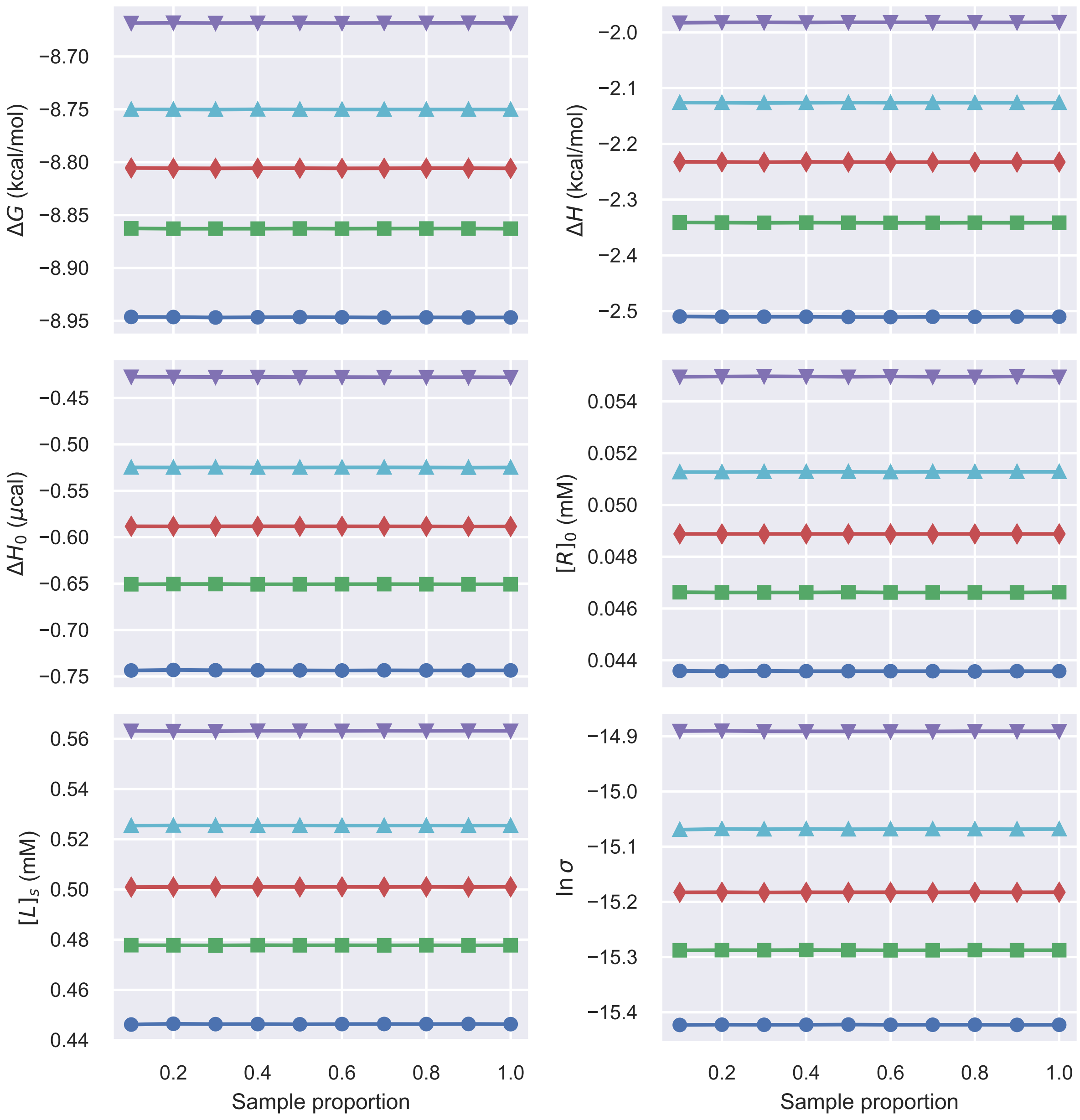

Mgp5EDTAp05b

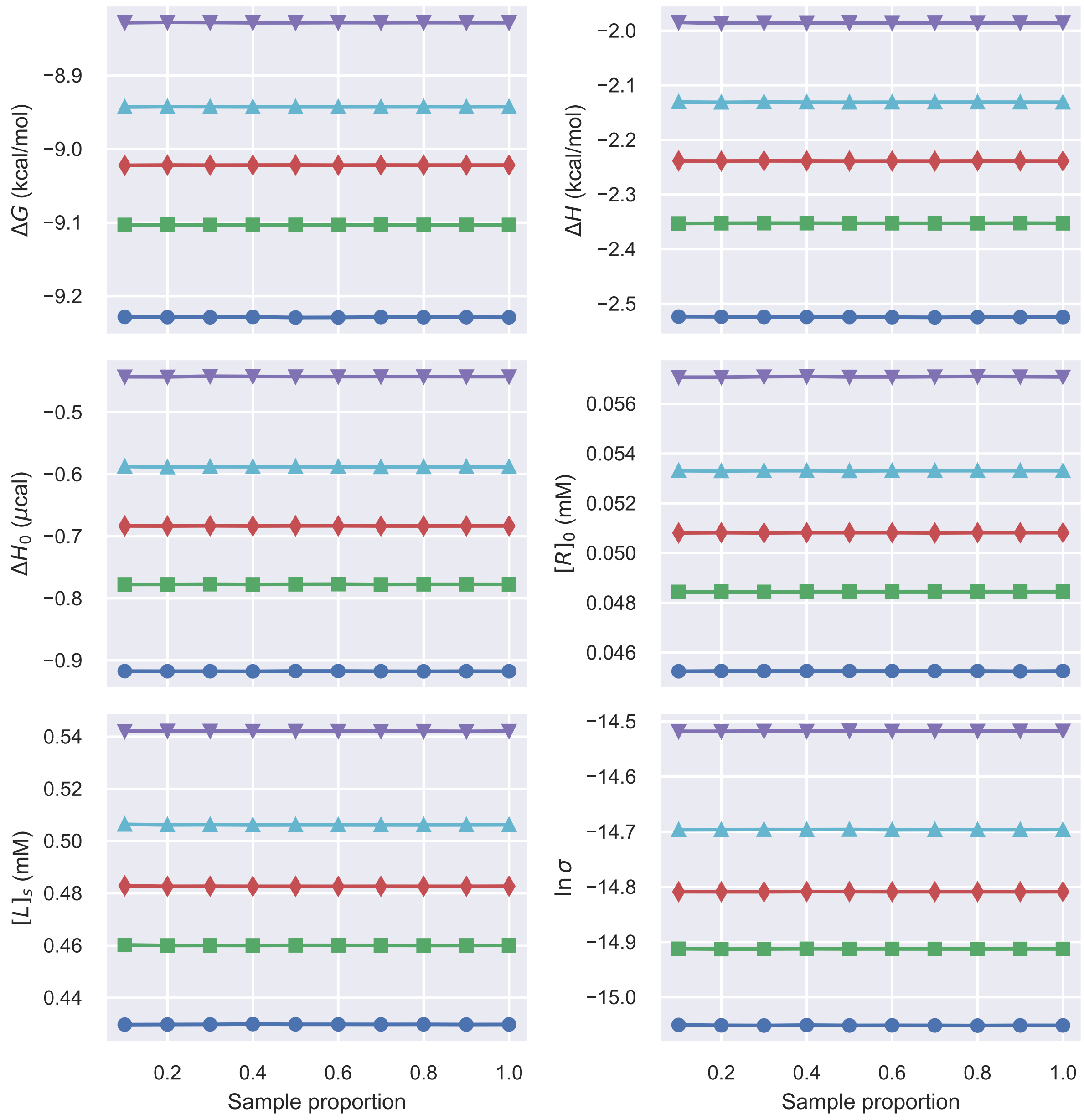

Mgp5EDTAp05c

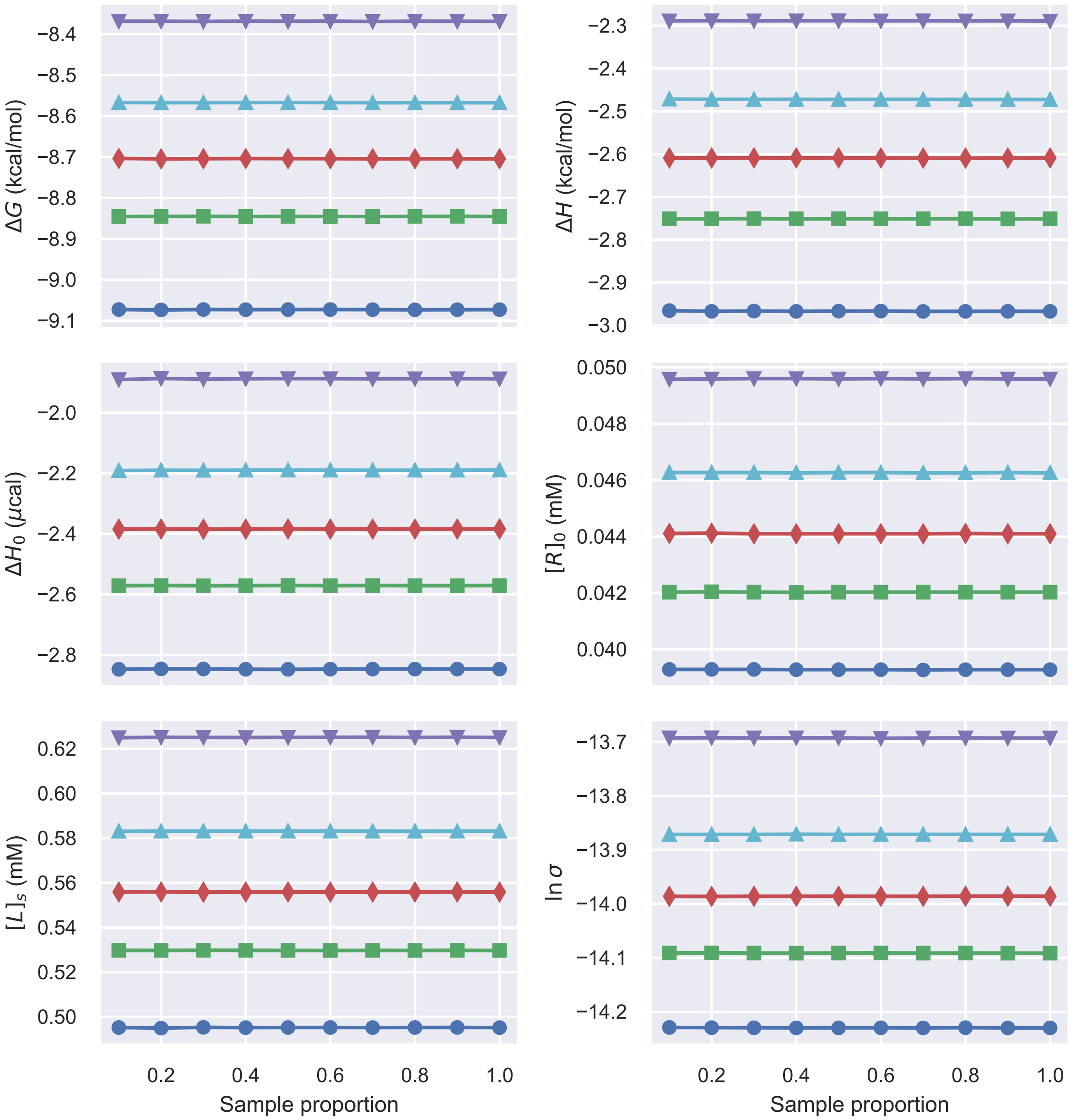

Mgp5EDTAp05e

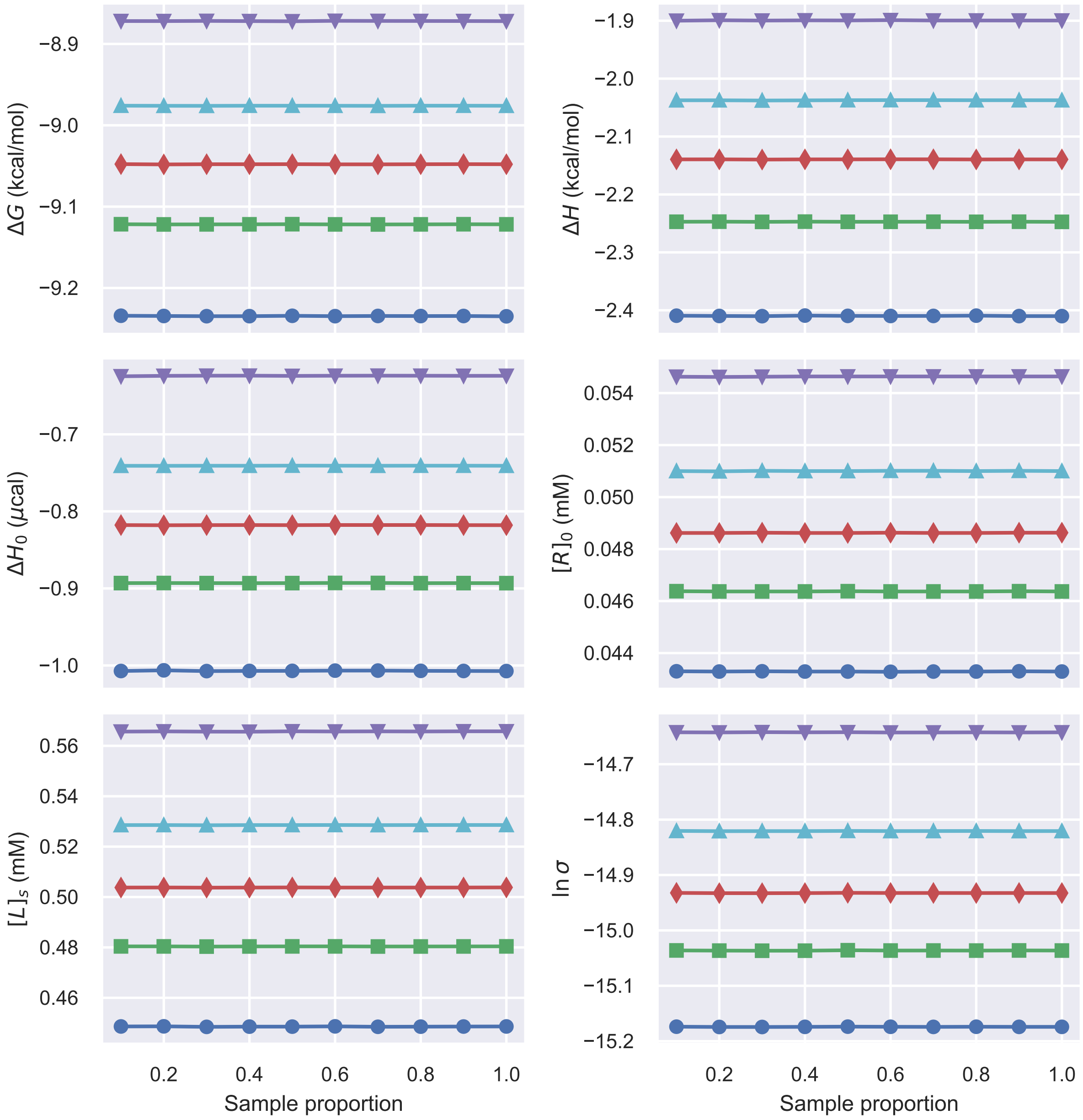

Mgp5EDTAp05f

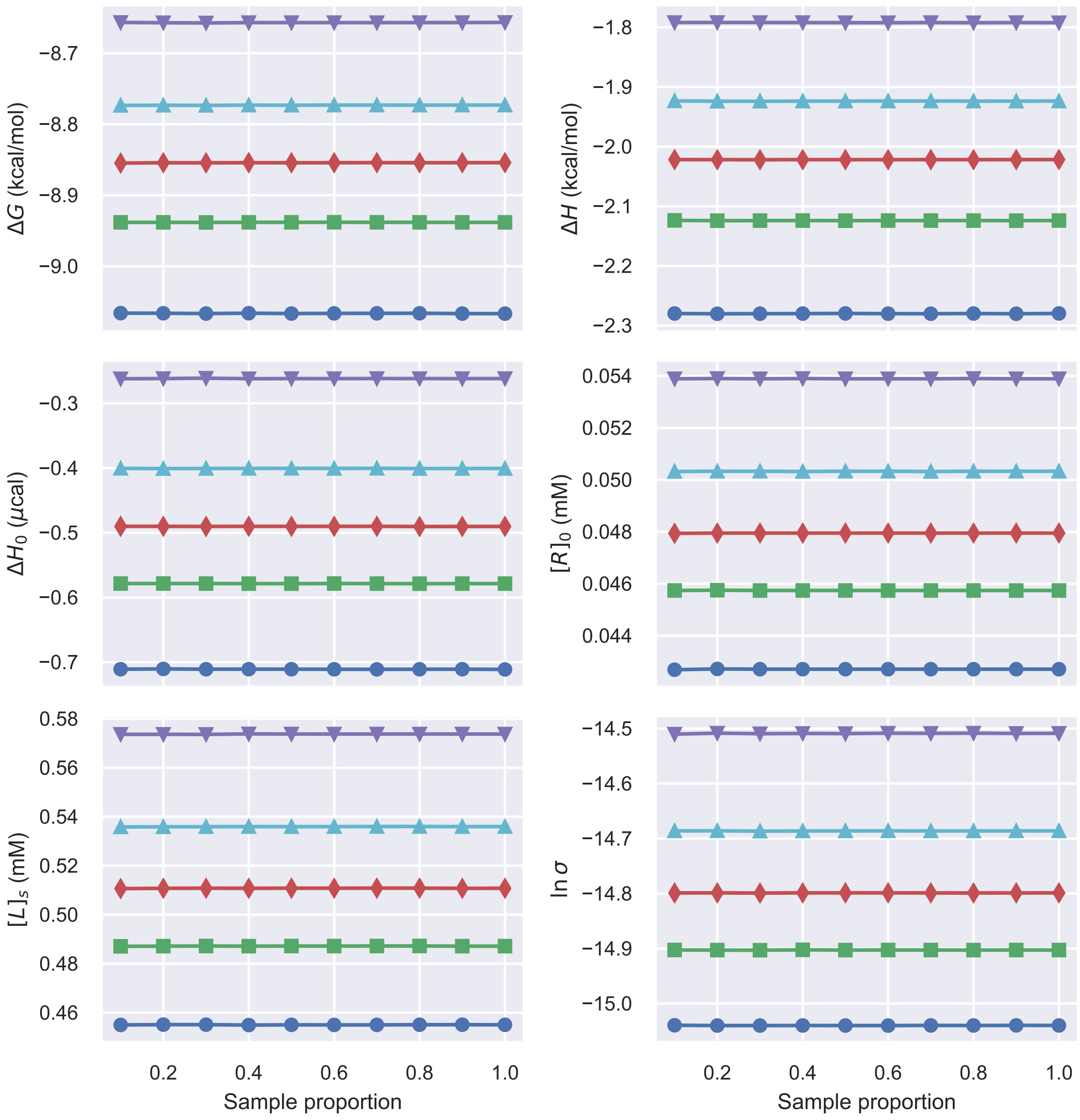

Mgp5EDTAp05g

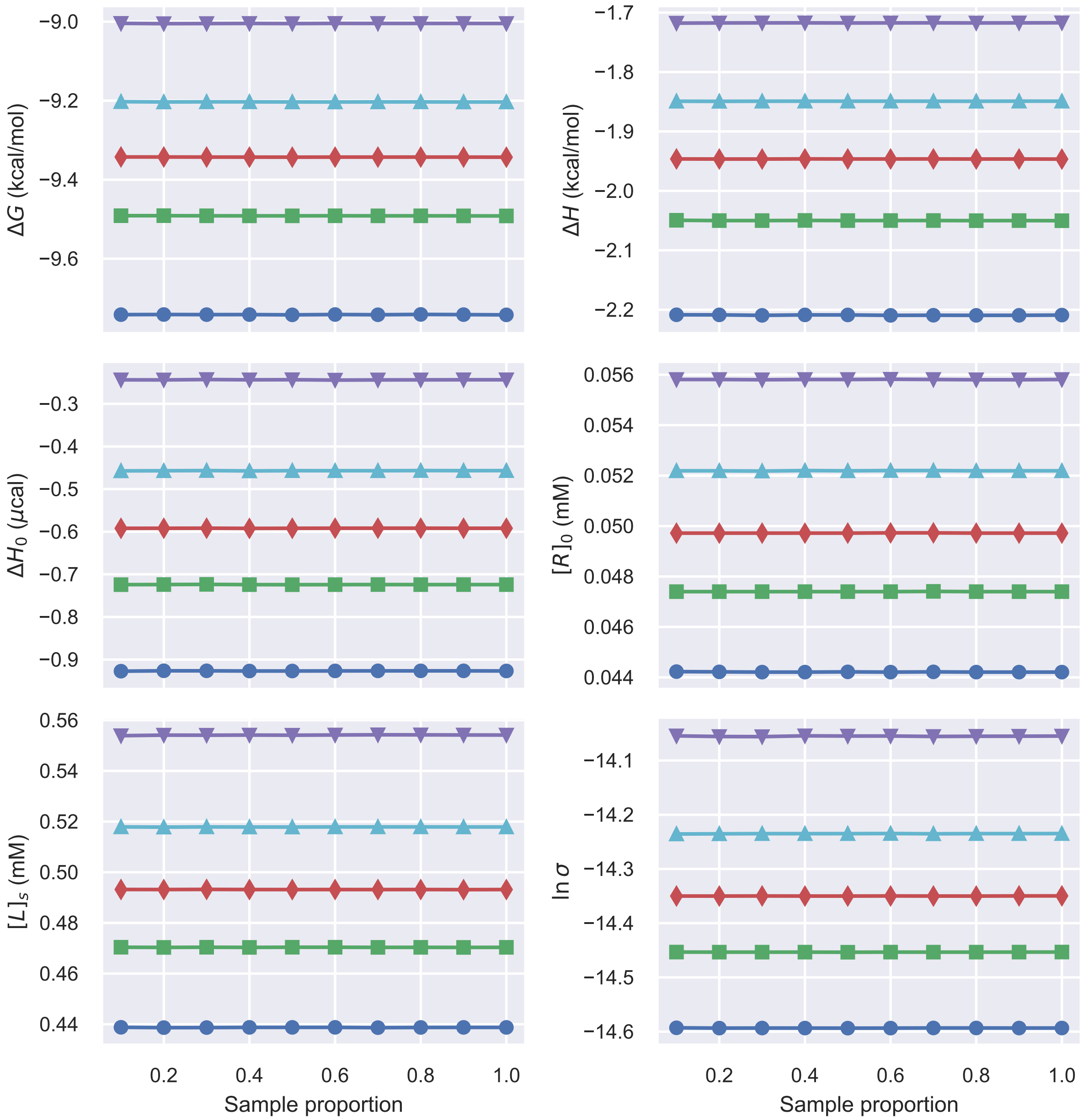

Mgp5EDTAp05h

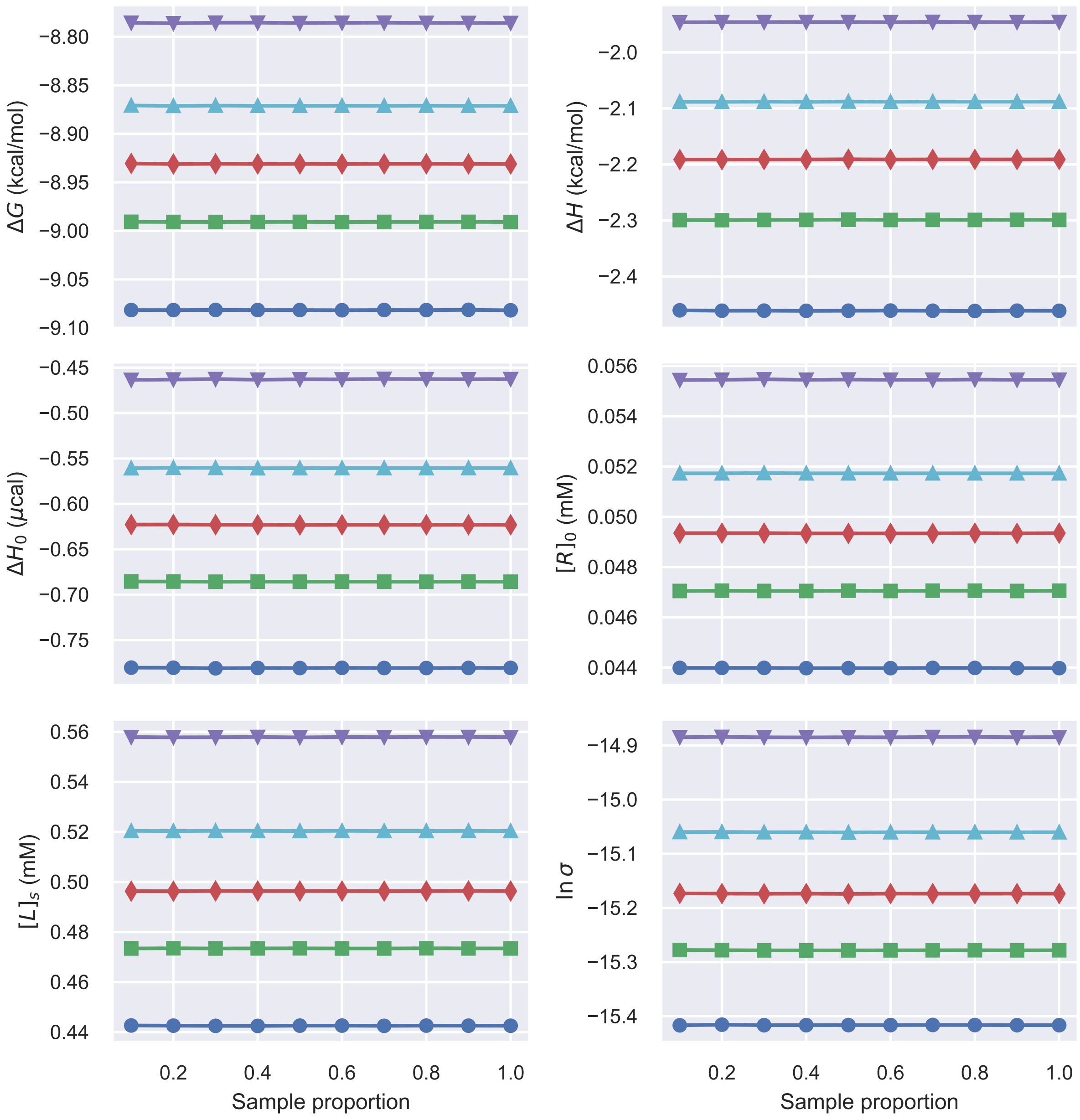

Mgp5EDTAp05i

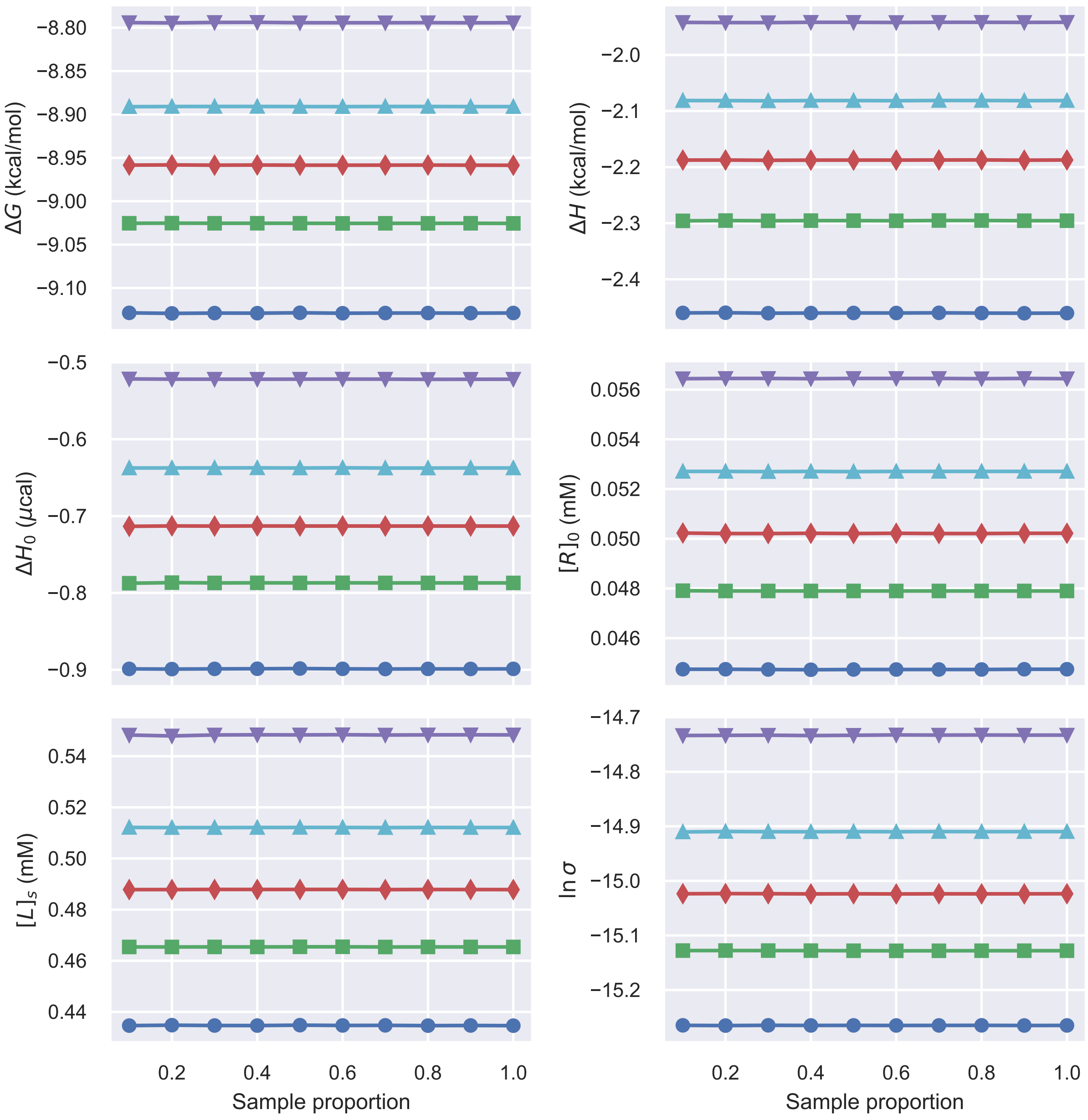

Mgp5EDTAp05j

Figure S4. Uncertainty validation of the simulation dataset at low error of 5%.

The predicted rate (%) of CIs containing the true values were plotted against the observed rate (%) for Bayesian credible intervals (blue leftward triangles), nonlinear least squares confidence intervals (red circles), and nonlinear least squares confidence intervals with error propagation (cyan downward triangles). Error bars of Bayesian procedure, which were

standard deviations based on 100 bootstrapping samples, were too small to be visible.

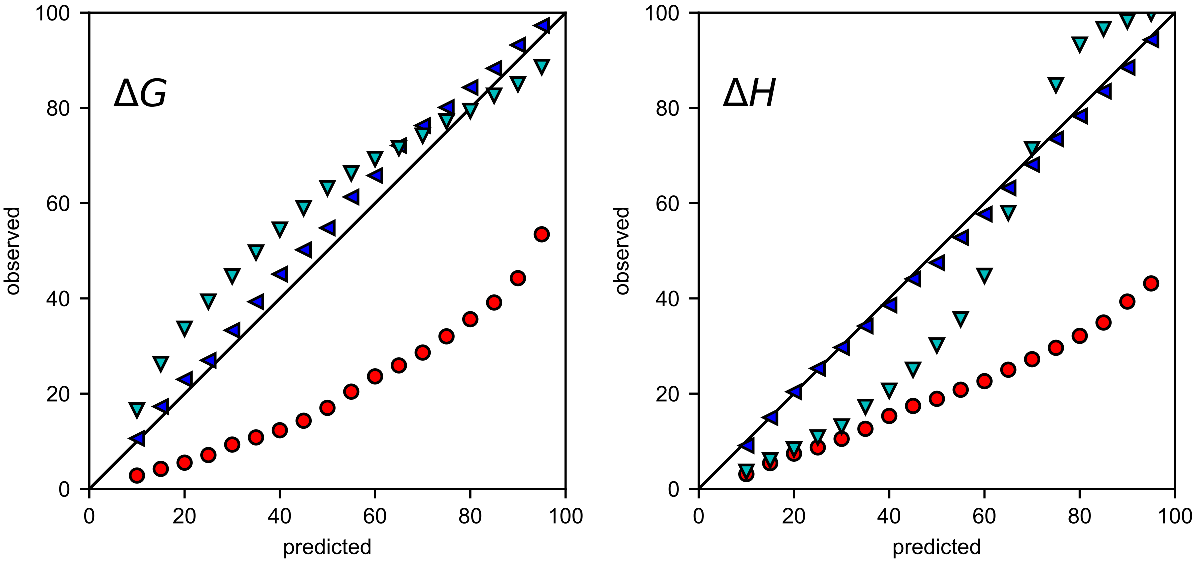

Supplement: Supplementary file 1 [file ijms-24-15074-s001.zip › ijms-2657944-supplementary.pdf]
